# Nasal lavage natural killer cell function is suppressed in smokers after live attenuated influenza virus

**DOI:** 10.1186/1465-9921-12-102

**Published:** 2011-08-04

**Authors:** Katherine M Horvath, Margaret Herbst, Haibo Zhou, Hongtao Zhang, Terry L Noah, Ilona Jaspers

**Affiliations:** 1 Curriculum in Toxicology, The University of North Carolina at Chapel Hill, Chapel Hill, NC 27599-7127, USA; 2 Department of Pediatrics, The University of North Carolina at Chapel Hill, Chapel Hill, NC 27599-7127, USA; 3 Center for Environmental Medicine, Asthma and Lung Biology, The University of North Carolina at Chapel Hill, Chapel Hill, NC 27599-7127, USA; 4 Department of Biostatistics, The University of North Carolina at Chapel Hill, Chapel Hill, NC 27599-7127, USA

## Abstract

**Background:**

Modified function of immune cells in nasal secretions may play a role in the enhanced susceptibility to respiratory viruses that is seen in smokers. Innate immune cells in nasal secretions have largely been characterized by cellular differentials using morphologic criteria alone, which have successfully identified neutrophils as a significant cell population within nasal lavage fluid (NLF) cells. However, flow cytometry may be a superior method to fully characterize NLF immune cells. We therefore characterized immune cells in NLF by flow cytometry, determined the effects of live attenuated influenza virus (LAIV) on NLF and peripheral blood immune cells, and compared responses in samples obtained from smokers and nonsmokers.

**Methods:**

In a prospective observational study, we characterized immune cells in NLF of nonsmokers at baseline using flow cytometry and immunohistochemistry. Nonsmokers and smokers were inoculated with LAIV on day 0 and serial nasal lavages were collected on days 1-4 and day 9 post-LAIV. LAIV-induced changes of NLF cells were characterized using flow cytometry. Cell-free NLF was analyzed for immune mediators by bioassay. Peripheral blood natural killer (NK) cells from nonsmokers and smokers at baseline were stimulated *in vitro *with LAIV followed by flow cytometric and mediator analyses.

**Results:**

CD45(+)CD56(-)CD16(+) neutrophils and CD45(+)CD56(+) NK cells comprised median 4.62% (range 0.33-14.52) and 23.27% (18.29-33.97), respectively, of non-squamous NLF cells in nonsmokers at baseline. LAIV did not induce changes in total NK cell or neutrophil percentages in either nonsmokers or smokers. Following LAIV inoculation, CD16(+) NK cell percentages and granzyme B levels increased in nonsmokers, and these effects were suppressed in smokers. LAIV inoculation enhanced expression of activating receptor NKG2D and chemokine receptor CXCR3 on peripheral blood NK cells from both nonsmokers and smokers *in vitro *but did not induce changes in CD16(+) NK cells or granzyme B activity in either group.

**Conclusions:**

These data are the first to identify NK cells as a major immune cell type in the NLF cell population and demonstrate that mucosal NK cell cytotoxic function is suppressed in smokers following LAIV. Altered NK cell function in smokers suggests a potential mechanism that may enhance susceptibility to respiratory viruses.

## Background

The nasal mucosa is the first site within the respiratory system to be exposed to pollutants and inhaled viral pathogens, including influenza. Therefore, nasal immune cells are likely to play important roles in early innate immune responses to these environmental stimuli. While macrophages and dendritic cells (DC)s have been identified in the nasal submucosa [1], and neutrophils have been identified in the nasal cavity [2], the overall immune cell populations within the nasal cavity have not been fully characterized. To phenotype nasal lavage fluid (NLF) cells, many researchers use cell differential analysis of cytocentrifuge slides stained with hematoxylin and eosin (H&E). Granulocytes are the easiest leukocytes to identify with H&E staining due to their polymorphic nuclei and are distinguished based on cytoplasmic staining: neutrophils have pale cytoplasm, eosinophils have a red granular cytoplasm, and basophils have a purple granular cytoplasm [3]. T or B lymphocytes are smaller cells with dark, dense nuclei and little cytoplasm [3]. Natural killer (NK) cells are larger lymphocytes with a pale cytoplasm and are difficult to distinguish due to a lack of specific cellular morphology. In fact, NK cells appear similar to macrophages or monocytes after H&E staining [3]. As a result, neutrophils, basophils, and eosinophils, but not NK cells, have been identified in NLF using cell differentials with H&E staining [4-6]. As an alternative to H&E staining, flow cytometry can be used to positively identify leukocytes in NLF. Flow cytometry has previously identified neutrophils in the NLF using CD16 expression [7,8] but expression of CD56, the classical NK cell marker, has not been used to positively identify NK cells in NLF. However, flow cytometric analysis has positively identified CD56(+) NK cells as well as CD3(+) T lymphocytes and HLA-DR(+) alveolar macrophages in the bronchoalveolar lavage of lung transplant recipients [9]. Thus, NK cells have been identified in the airways of humans, [10] but whether NK cells are present in the nasal cavity and how they could function as a guard against inhaled pollutants or pathogens is not known.

Influenza infection induces the recruitment of immune cells into the lung, including NK cells [10]. NK cells perform essential functions such as killing virus-infected epithelial cells and secreting cytokines to regulate innate and adaptive immune responses [11]. CD16, an FC receptor that induces antibody-dependent cell mediated cytotoxicity [12], is classic marker identifying neutrophils [13] but is also expressed on cytotoxic NK cells [11]. CD16^+ ^cytotoxic NK cells also have dim CD56 expression and release cytotoxic granules containing perforin and granzymes to induce apoptosis in influenza infected cells [14]. In contrast, CD16(-) NK cells have bright CD56 expression and are considered "cytokine-secreting" NK cells as they secrete IFNγ that matures dendritic cells (DCs) [14]. NK cell activation during influenza infection is dependent upon secretion of cytokines and chemokines such as "regulated upon activation, normal T cell expressed and secreted" (RANTES) and interferon gamma-induced protein 10 kDa (IP-10) from cells in the respiratory mucosa [15,16] through binding to chemokine receptors CCR5 and CXCR3, respectively. Upon activation by pathogens and inflammatory mediators such as Type I IFNs, IL-12, and IL-15, NK cells can become more cytotoxic as they reduce CD56 expression and acquire CD16 expression [17]. NK cells can also be activated by receptor mediated interactions. NK cells express many activating receptors, including NK cell activating receptor 2D (NKG2D), which recognizes ligands induced during cellular stress. As such, binding of ligands to the NKG2D receptor on NK cells enhances NK cell cytokine production as well as cytotoxic activity[16]. In this manner, influenza infection in the respiratory tract may activate NK cells either through modification of activating surface and chemokine receptors or enrichment of the NK cell cytokine microenvironment. However, the role of NK cells in antiviral responses to influenza infection within the nasal passages has yet to be determined.

Airborne pollutants, such as cigarette smoke, have been shown to increase susceptibility to respiratory viral infections, including influenza [18-20]]. We have recently demonstrated that smokers' nasal epithelial cells have modified responses to influenza infections both *in vivo *[2] and *in vitro *[21] resulting in increased markers of influenza infection. Because NK cells can control and regulate viral infections via killing of infected respiratory epithelial cells, altered NK cell functions in smokers could contribute to enhanced influenza infections. Although smoking has been shown to suppress peripheral NK cell activity *ex vivo *[22-26], the effects of smoking on respiratory NK cell functions are unknown [24].

Our goals were to 1) phenotype immune cells in the nasal passages using flow cytometry, 2) determine the presence and function of these cells in the context of a viral infection, and 3) assess the effects of cigarette smoke exposure on nasal immune cell function. Nonsmokers and smokers were inoculated with the live attenuated influenza virus (LAIV) vaccine, similar to our previous study [2]. Serial nasal lavages were used to compare immune cell, and specifically NK cell, function in the nasal cavity of smokers and nonsmokers. We also compared peripheral blood NK cell responses to LAIV in nonsmokers and smokers. Our results show that NK cells are present in the NLF, NK cells in the NLF change after inoculation with LAIV, and NK cell responses are modified in smokers following LAIV.

## Methods

### Effect of LAIV on Nasal Immune Responses: Study Design

This was a prospective longitudinal study comparing responses to LAIV between cohorts of healthy young adult smokers and nonsmokers. The study design was as described before [2]. Baseline measurements were done at a screening visit and Day 0. On Day 0 subjects received a standard nasal inoculum of the 2008-2009 formulation of LAIV (FluMist^®^, MedImmune, Gaithersburg, MD; administered by study nurse according to manufacturer's instructions) in both nostrils, then returned on Days 1, 2, 3, 4, and 9 post-LAIV for serial nasal lavages. Subject exposure history questionnaires and urine cotinine levels were used to estimate cigarette smoke exposure.

### Study subjects

Subjects were identified as described before [2] and included healthy young adults between 18-35 years old in two groups: Group 1 = nonsmokers not regularly exposed to secondhand smoke (nonsmokers who neither live nor work with active smokers) and Group 2 = self-described active cigarette smokers. Informed consent was obtained from all subjects and the protocol was approved by the UNC Biomedical Institutional Review Board. Exclusion criteria were as described before [2]. Table 1 details demographic and smoke exposure characteristics of the subjects completing the study. Nonsmokers and smokers did not differ significantly for age, BMI, or gender. One of 14 enrolled nonsmoker subjects and 5 of 20 enrolled smoker subjects dropped out before completion of the study. Two smoker nasal lavage fluid sample sets were compromised by freezer malfunction and therefore were not included in the analysis. Self-described smokers had significantly higher secondhand smoke exposure and urine cotinine values compared to nonsmokers. No serious adverse events occurred among subjects completing the protocol.

**Table 1:** Subject characteristics and tobacco smoke exposure

	Nonsmoker (n = 13)	Smoker (n = 13)
**Age (yr)**	25.3 ± 1.0	23.5 ± 1.2
**Gender**	5M/8F	7M/6F
**BMI**	25.1 ± 1.3	28.2 ± 2.3
**Daily exposure**		
**Cigarettes smoked^1^**	NA	8.5 ± 1.6
**Urine cotinine^2^**	0.0 ± 0.0	19.5 ± 7.1***

^1 ^Data shown as mean ± SEM for cigarettes smoked. Data were averaged from each subject's self reported estimates for study days 0 through 9.

^2 ^Data shown as mean ± SEM for mg cotinine (× 100)/mg creatinine. Data were obtained using screen urine values.

*** P < .0001 vs. nonsmoker

### Nasal lavage

Nasal lavage was performed using a method we have previously described [2,27]. In brief, 4 ml of saline was sprayed into each nostril in 100 ul repetitive sprays followed by periodic forceful expulsion of fluid into a collection cup. Fluid from both nostrils was pooled. The NLF was filtered using 40 μm cell strainer (BDBiosciences, San Jose, CA), and the NLF filtrate was pelleted by centrifugation. Prior to dithiothreitol (DTT) treatment, cell-free NLF was stored in aliquots at - 80°C until used in mediator assays. Contents of the cell strainer were treated with 1:20 DTT concentrate solution (Sputolysin^®^, EMD Chemicals, Gibbstown, NJ) in HBSS. Filtered cells were combined with DTT treated cells to comprise the total NLF cell pellet. Cytocentrifuge slides were stained for differential cell counts and immunohistochemistry as described below The NLF cell pellet was processed for flow cytometry as described below to identify and quantify immune cells.

### NLF Cell Differentials and Immunohistochemistry

Cytocentrifuge slides were prepared, fixed, and stained using a modified Wright stain for differential cell counts. For immunohistochemistry, cytocentrifuge slides were fixed with ice-cold methanol, washed with TBS and blocked with Powerblock (Biogenex, San Ramon, CA) for 1 hr at room temperature. Slides were then incubated with the following primary antibodies: mouse anti-human CD56 antibody (MAB24081 RnD, Minneapolis, MN) or mouse anti-human perforin (BD Biosciences) overnight at 4°C. The slides were washed with TBS. Following incubation with an HRP (horse radish peroxidase)-conjugated secondary antibody, samples were washed with TBS and evaluated under light microscopy. Nonspecific staining was assessed by omitting the target-specific primary antibody. A full list of all antibodies used for immunohistochemistry and flow cytometry is located in Table 2.

**Table 2:** Antibodies Used

Antibody [Reference]	Isotype Control	Dilution	Company	Clone	Catalog Number
**Anti-human CD56 **[48]	Mouse IgG2b, κ	1:100	RnD Systems	301021	MAB24081
**Anti-human perforin **[49]	Mouse IgG2b, κ	1:100	BDBiosciences	δG9	556434
**APC anti-human NKG2D **[50]	Mouse IgG1, κ	1:10	Biolegend	1D11	320808
**APC-Cy7 anti-human CD45 **[51]	Mouse IgG1, κ	1:20	BDBiosciences	2D1	557833
**FITC anti-human CD16 **[52]	Mouse IgG1, κ	1:10	Beckman Coulter	3G8	IM0814U
**Pacific Blue anti-human CD14 **[53]	Mouse IgG2a, κ	1:20	BDBiosciences	M5E2	558121
**PE anti-human CD56 **[54]	Mouse IgG1, κ	1:10	BDBiosciences	B159	555516
**PE-Cy5 anti-human CD4 **[55]	Mouse IgG1, κ	1:10	BDBiosciences	RPA-T4	555348
**PerCP anti-human CD3 **[56]	Mouse IgG1, κ	1:10	BDBiosciences	SK7	347344
**PerCP/Cy5.5 anti-human CXCR3**[57]	Mouse IgG1, κ	1:40	Biolegend	TG1/CXCR3	334905
**Biotinylated horse anti-** **mouse IgG **[58]	Horse	1:200	Vector Labs		BA-2000
**APC mouse IgG1, κ**		1:100	BDBiosciences		555751
**FITC mouse IgG1, κ**		1:100	BDBiosciences		555748
**Pacific Blue mouse IgG2a, κ**		1:100	BDBiosciences		558118
**PE mouse IgG1, κ**		1:100	ebioscience		12-4714-81
**PE-Cy5 mouse IgG1, κ**		1:100	BDBiosciences		555750
**PerCP mouse IgG1, κ**		1:100	BDBiosciences		550672

### Ex vivo NLF Cell Flow cytometry

The NLF cell pellet was suspended in flow cytometry buffer (PBS, 0.09% sodium azide, 1% heat inactivated FBS) and stained with antibodies to CD16 FITC (Beckman Coulter, Brea, CA), CD14 Pacific Blue (Biolegend, San Diego, CA) CD56 PE, CD4 Pe-Cy5, CD3 PerCP, CD45 APC-Cy7 (BD Biosciences) for 20 minutes at room temperature in the dark. Cells were washed with flow cytometry buffer, resuspended in 0.5% paraformaldehyde, and stored at 4°C in the dark. Samples were acquired within 24 hrs on a BDLSRII flow cytometer (BD Biosciences). Isotype-matched single color controls were used to control for nonspecific staining and to set analysis gates.

### NLF mediator and urine cotinine assays

NLF granzyme B activity was measured using a SensiZyme Granzyme B Activity kit (Sigma, St. Louis, MO). Urine cotinine was measured by ELISA (Bio-Quant, Inc., San Diego, CA) and expressed as a ratio to creatinine, measured by a colorimetric assay (Oxford Biomedical Research, Rochester Hills, MI).

### In vitro NK Cell Stimulation Assays

Peripheral blood NK cells were isolated from peripheral blood mononuclear cells (PBMC) from nonsmokers and smokers at baseline and stimulated *in vitro *with the 2008-2009 strain of LAIV. PBMC were isolated from nonsmokers and smokers using Lymphoprep™ (Axis-Shield, Oslo, Norway). NK cells were isolated from PBMC as described before [28] using the RosetteSep^® ^Human NK Cell Enrichment Cocktail (Stemcell, Vancouver, British Columbia). In brief, PBMC were incubated with a 1:20 dilution of the antibody enrichment cocktail in RPMI complete media [10% FBS, l-glutamine (Invitrogen), and penicillin:streptomycin (Invitrogen)] and washed red blood cells (RBC) in a ratio of 100:1 RBC to PBMC. Cells were incubated at room temperature for 20 minutes with gentle shaking. Cells were washed with equal volume RPMI complete media, layered on Lymphoprep™ gradient, and centrifuged at 800 g for 20 minutes at room temperature. Non-NK PMBC pelleted with RBC and NK cells were located in the interphase. Enriched NK cells were isolated and washed with RPMI. 1 × 10^5 ^NK cells were stimulated with 0.1 ul (2.56 HAU) LAIV (see below) in 100 ul of RPMI complete media for 24 h at 32°C in 5% CO_2_. NK cells were centrifuged at 500 g for 5 min and processed for flow cytometry as described below. Cell-free supernatant was aliquoted and stored at -80°C for assessment of granzyme B activity as described below.

### LAIV propagation in MDCK cells in vitro

The 2008-2009 LAIV strain was propagated in Madin-Darby Canine Kidney (MDCK) epithelial cells *in vitro*. 0.05 MOI LAIV stimulated 90% confluent MDCK cells in serum free DMEM media supplemented with pen-strep, l-glutamine and 0.2% trypsin without EDTA (all Invitrogen) and incubated for 48 h at 32°C in 5% CO_2_. The cell supernatant was gently aspirated and combined with 10% fetal bovine serum (Invitrogen) to inactivate trypsin. Cells and debris were pelleted by centrifugation at 500 g for 10 minutes. Cell free supernatant was concentrated by centrifugation with an Amicon Ultra-15 Centrifugal Unit (Millipore, Billerica, MA) using a 100,000 molecular weight cutoff. Smaller proteins (cytokines) fall through the filter whereas larger viruses (LAIV) are collected. To generate a vehicle control, MDCK cells were mock-infected with media and the supernatant was processed in the same fashion. Concentrated LAIV was aliquoted and stored at -80°C until use. LAIV was titered using a hemagglutination assay as described before [29]. The titer for the propagated virus was 25.6 HAU (hemagglutinin units)/ul, which was 8× higher than the original 2008-2009 LAIV strain (data not shown). MDCK-propagated LAIV was used in all *in vitro *assays.

### Peripheral NK Cell Flow Cytometry

NK cells were washed and resuspended in flow cytometry buffer and stained with antibodies to CD16 FITC (Beckman Coulter), CD56 PE, CD3 APC-Cy7 (BD Biosciences), CXCR3 and NKG2D (Biolegend) for 20 minutes at room temperature. Processing was completed as described above in the *ex vivo *NLF flow cytometry section.

### Peripheral NK Cell Granzyme B Activity

Granzyme B activity from the *in vitro *LAIV NK cell stimulation was quantified as described before [30]. Briefly, NK cell supernatants were combined 1:1 with 50 mM HEPES pH 7.5, 0.1% CHAPS, 10% sucrose, (all Sigma) and 400 uM colorimetric granzyme B substrate I (EMD4Biosciences, Merck, Darmstadt, Germany). Supernatants were incubated at 37°C in 5% CO_2 _for 24 h. Absorbance was read using a plate reader at 405 nm wavelength. A standard curve of granzyme B (Sigma) with 1:1 serial dilutions was used to calculate specific activity.

### Statistical Analysis

Baseline differences between NLF cell populations were determined using a Mann-Whitney U test. The effects of smoking status on NLF responses to LAIV were analyzed using a Kruskal-Wallis One-way ANOVA followed by Bonferroni's posttest to determine differences on individual days. An area under the curve (AUC) analysis followed by a Kruskal-Wallis One-way ANOVA was used as previously described [2] to determine the effects of smoking status on total immune NLF responses to LAIV. Within nonsmoker and smoker groups, Wilcoxon matched pairs tests were used to determine the effects of LAIV on peripheral NK cells *in vitro*. Data were shown as mean ± SEM or median (interquartile range).

## Results

### Characterizing immune cells in NLF at baseline in nonsmokers

**Figure 1:**
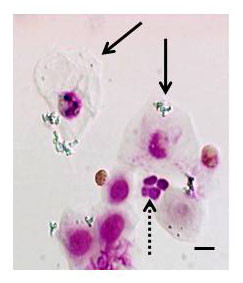
**Nasal Lavage Cells**. NLF cells from nonsmokers at baseline were stained with hematoxylin and eosin. Squamous epithelial cells (black arrows) and neutrophils (dashed arrows) can be positively identified. n = 12. A representative image is shown. Bar = 10 μm.

**Figure 2:**
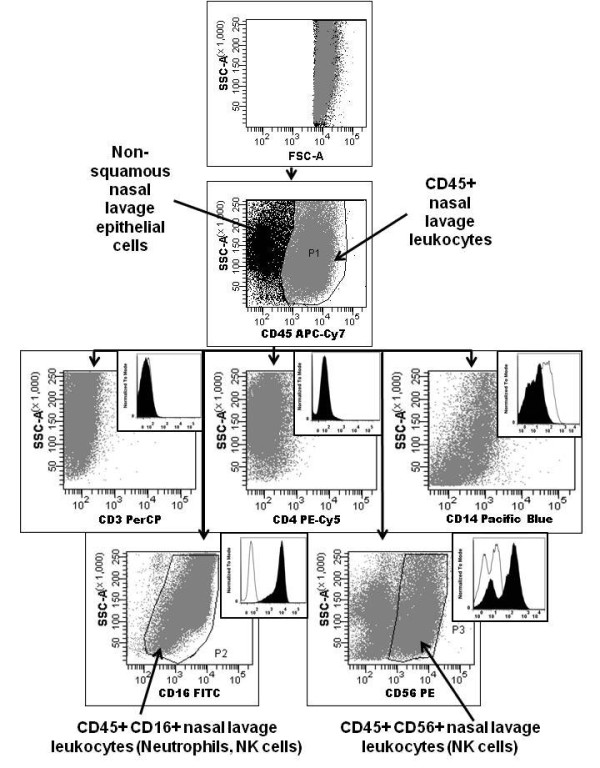
**Nasal Lavage Immune Cell Flow Cytometry**. NLF cells were collected from nonsmokers at baseline and analyzed by flow cytometry for leukocyte markers. The majority of NLF squamous epithelial cells are gated out by FSC and SSC settings. CD45+ NLF leukocytes are identified in NLF cells. CD45+ NLF cells (P1) are negative for surface markers CD3 (T lymphocytes) CD4 (Helper T lymphocytes), and CD14 (monocytes). CD45+ NLF cells contained populations positive for CD16 (neutrophils, NK cells, P2) and CD56 (NK cells, P3). A representative image at baseline is shown. Open histograms: isotype matched control, P1. Shaded histograms: surface markers, P1.

A representative cellular differential of NLF cells was pictured in Figure 1. Squamous cells (black arrows) in the nasal lavage comprised 52.0% (37.3-62.5) of total NLF cells in nonsmokers at baseline. Neutrophils (dashed arrows) could also be identified by morphology in the nasal lavage and comprise 7.7% (2.1-16.5) of non-squamous NLF cells. We used flow cytometry to positively identify other non-squamous NLF cells. As shown in Figure 2, forward scatter (FSC) and side scatter (SSC) settings eliminated squamous epithelial cells from the view. Negative expression of CD45, a marker that stains only leukocytes, was used to discriminate between NLF non-squamous epithelial cells and NLF leukocytes of similar size. Figure 2 showed further analysis of CD45(+) NLF cells stained for surface marker expression of CD3 (T lymphocytes), CD4 (T helper lymphocyte) CD14 (monocytes), CD16 (neutrophils), and CD56 (NK cells). These data showed that of the markers tested, only substantial subpopulations positive for CD16 and CD56 were identified in CD45^+ ^NLF cells.

**Figure 3:**
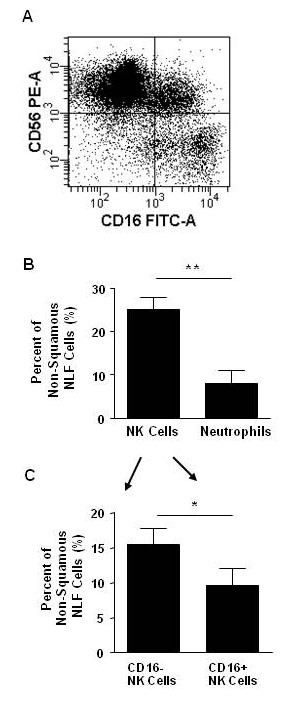
**Characterization of NK Cells in the Nasal Lavage by Flow Cytometry**. NK cells were identified in CD45+ NLF cell populations of nonsmokers at baseline. A) Representative flow cytometric plot depicting NK cells (CD56+) and neutrophils (CD56-CD16+). B) Percentages of total NK cells are greater than neutrophils in non-squamous NLF cell populations. C) Percentages of CD16- NK cells are greater than percentages of CD16+ NK cells in non-squamous NLF cell populations. **p < 0.01, * p < 0.05. Nonsmoker n = 11.

To further characterize the immune phenotype of NLF cells, we focused our analysis on CD16 and CD56. In Figure 3A, a representative flow cytometric dot plot showed CD16 and CD56 co-expression on CD45(+) cells. Distinct CD56(+) NK cell (upper left quadrant) and CD56(-)CD16(+) neutrophil (lower right quadrant) populations were depicted. Also, CD16(-) NK cells appeared to have "brighter" CD56 expression compared to their CD56(+)CD16(+) NK cell counterparts. Figure 3B indicated that NK cells comprised a greater percentage [23.3% (18.3-34.0)] compared to neutrophils [4.6% (0.3-14.5)] of non-squamous NLF cells (p < 0.01). Percentages of cytokine-secreting CD16(-) NK cells [13.0% (10.4-20.3)] were greater than percentages of cytotoxic CD16(+) NK cells [8.8% (3.6-12.7)] in nonsmokers at baseline as shown in Figure 3C (p < 0.05). None of the CD45(+) leukocytes in the nasal lavage were CD3(+), indicating that the CD56(+) cells were not natural killer T (NKT) cells.

**Figure 4:**
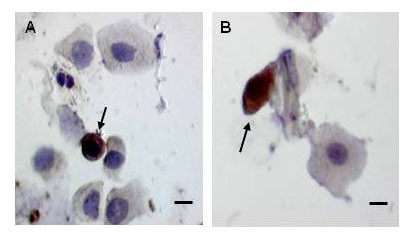
**Characterization of NK cells in the Nasal Lavage by Immunohistochemistry**. NLF cells were characterized using immunohistochemistry. A) NLF cells are stained with anti-CD56-HRP to identify NK cells and B) NLF cells are stained with anti-perforin-HRP to identify cytotoxic NK cells. Bar = 10 μm.

We used immunohistochemistry to confirm the presence of NK cells in the NLF. Positive immunohistochemical staining for CD56 identified NK cells in Figure 4A. Cytotoxic NK cells were identified by immunohistochemical staining for perforin, a cytotoxic granule expressed in cytotoxic T lymphocytes and NK cells, as shown in Figure 4B.

### Comparison of smokers vs nonsmokers after LAIV

Smoker and nonsmoker groups did not differ by age, BMI, or gender (Table 1). As expected, smokers had significantly greater average urine cotinine levels than nonsmokers, indicating that the smokers continued to actively smoke during the study period.

**Figure 5:**
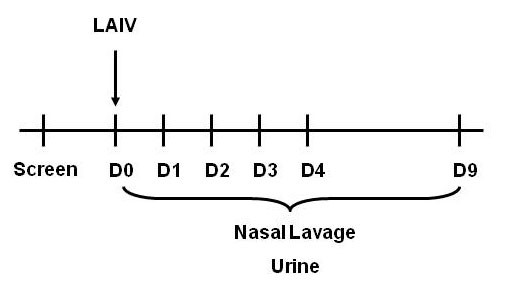
**Schematic of LAIV Study Design**. Nonsmokers and smokers were given a baseline nasal lavage followed by inoculation with LAIV on Day 0. Serial nasal lavages were obtained on Days 1-4 and again on Day 9. Urine was collected throughout to study to measure cotinine, a metabolite of nicotine, as a marker of cigarette smoke exposure.

**Figure 6:**
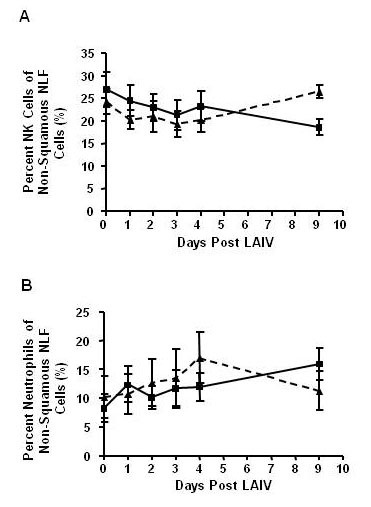
**LAIV Does Not Significantly Affect Total NK Cell or Neutrophil Percentages in Nonsmokers or Smokers**. Using flow cytometry we identified NK cells and neutrophils in nonsmoker and smoker non-squamous NLF cells after LAIV inoculation. Neither A) NK cell nor B) neutrophil percentages in total NLF cells change following LAIV in either group. Nonsmokers n = 12 (■, solid line), smokers n = 9 (▲, dashed line).

**Figure 7:**
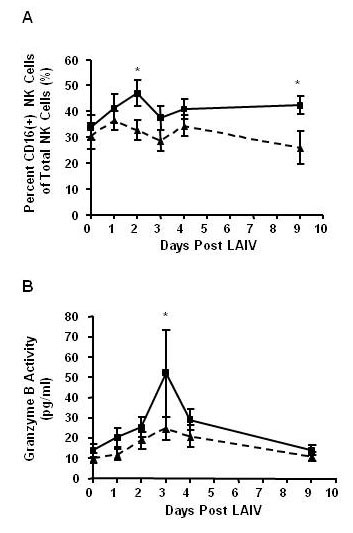
**Cytotoxic NK Cell Activity is Suppressed in Smokers Following LAIV**. Cytotoxic NK cell percentages and activity were analyzed in NLF of nonsmokers and smokers after LAIV inoculation. A) CD16+ cytotoxic NK cell percentages of total NK cells were decreased in the NLF of smokers following LAIV. Kruskal-Wallis p = 0.09, *p < 0.05 nonsmoker vs smoker posttest. Nonsmokers n = 12 (■, solid line), smokers n = 9 (▲, dashed line). B) Granzyme B activity was decreased in NLF of smokers following LAIV inoculation. Kruskal-Wallis p < 0.01, *p < 0.05 nonsmoker vs smoker post-test. Nonsmokers n = 13 (■, solid line), smokers n = 13 (▲, dashed line).

Using flow cytometry, percentages of NK cells and neutrophils were quantified in the non-squamous NLF cell population of nonsmokers and smokers before (Day 0) and after inoculation with LAIV (Days 1-4, 9). See schematic in Figure 5. There were no statistically significant differences in total NK cell percentages associated with either LAIV or smoking status (see Figure 6A). Total neutrophil percentages were also similar between groups and unchanged after LAIV (Figure 6B), which confirms our previous observations [2]. To further characterize NK phenotypes after LAIV, proportions of cytotoxic NK cells within the total NK cell population were determined by assessing CD16(+) expression on NK cells using flow cytometry and cytotoxic NK cell activity was determined by measuring granzyme B bioactivity in the NLF. CD16(+) NK cells increased by day 2 after LAIV in nonsmokers, but this increase was significantly blunted in smokers (Figure 7A). Similarly, the rise in granzyme B seen in nonsmokers by day 3 was significantly suppressed in smokers (Figure 7B); as well as an overall suppression of granzyme B response post LAIV in smokers, albeit not statistically significant (p = 0.09, Table 3).

**Table 3:** Total NLF granzyme B responses post LAIV.

	Nonsmokers (N = 13)	Smokers (N = 13)	P (Kruskal-Wallis)
**Granzyme B**	225.0 (68.7-519.3)	111.5 (14.9-310.0)	0.09

Data are shown as median (interquartile range). Area under NLF mediator quantity, Day 1-9 after LAIV inoculation.

To determine whether the effects of cigarette smoke exposure on NK cell function are evident systemically, peripheral blood NK cells were isolated from nonsmokers and smokers and stimulated *in vitro *with LAIV. Preliminary studies indicated that peripheral blood NK cells were CD45(+), as expected, so we excluded this marker from further analysis (data not shown).

**Figure 8:**
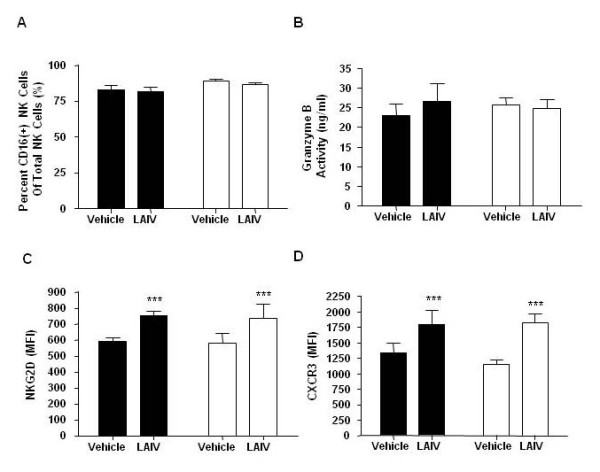
**Effect of LAIV On Peripheral NK Cell Activity**. Peripheral blood NK cells were isolated from nonsmokers and smokers and stimulated *in vitro *with LAIV. LAIV did not increase A) percent CD16+ cytotoxic NK cells or B) granzyme b activity in either nonsmokers or smokers. LAIV increased expression of C) NK cell activation receptor NKG2D and D) chemokine receptor CXCR3 in both nonsmokers and smokers. Nonsmokers n = 6 (black bars), smokers n = 6 (white bars). *** p < 0.001 vs vehicle control.

Percentages of peripheral cytotoxic CD16(+) NK cells were not altered by *in vitro *stimulation with LAIV in either nonsmokers or smokers (Figure 8A). Interestingly, peripheral blood cytotoxic CD16(+) NK cells composed a larger proportion of total NK cells [84.2% (79.3-90.4)] (Figure 8A) versus mucosal NK cell populations [28.3% (22.2-46.2)] in nonsmokers at baseline (Figure 7A) (p < 0.0001). LAIV did not induce granzyme B secretion in peripheral NK cells from smokers or nonsmokers (Figure 8B). To determine if LAIV stimulation *in vitro *can alter expression of peripheral NK cell activating and chemokine receptors (NKG2D and CXCR3), we assessed receptor expression by flow cytometry. LAIV increased NKG2D (Figure 8C) and CXCR3 (Figure 8D) expression on peripheral NK cells from both nonsmokers and smokers. However, there were no differences in peripheral NK cell receptor expression between smokers and nonsmokers.

## Discussion

Characterizing innate immune cells within the nasal passages is an important step in understanding how pre-existing conditions, such as smoking, affect anti-influenza responses in the respiratory epithelium. Using a model of *in vivo *human influenza infection [2] and *ex vivo *flow cytometric methodology, we observed that 1) NK cells are present in nasal secretions and constitute a significant portion of NLF immune cells, 2) the "normal" nasal NK cell response to LAIV involves an increase in activated cytotoxic NK cells, and 3) these LAIV-induced cytotoxic NK cell responses in are suppressed in smokers.

The identification of NK cells as a prominent immune cell type in NLF is, to our knowledge, a novel finding and suggests that the study of innate immune responses in the upper airways should take NK cells into account. The use of cell differentials alone to phenotype NLF cells has likely overlooked NK cells [4-6]. As shown in Figure 4, NK cells have non-descript morphologies and could be mistaken for NLF monocytes, macrophages or even basal epithelial cells. Using flow cytometry, other researchers have identified CD16(+) NLF cells. However, these CD16(+) cells were either classified as neutrophils [7] or the analysis gate was based on the relative size of a lymphocyte population [8], thus likely excluding NK cells. In our study, activated NK cells in NLF appeared to be of similar size and granularity as neutrophils as evidenced by the flow cytometric CD56 staining and SSC properties (see Figure 2). This is not surprising as both NK cells and neutrophils contain cytotoxic granules that should influence their SSC fluorescence. Figure 4 shows that NK cells positively identified in the NLF using immunohistochemistry are relatively large cells compared to T lymphocytes [3] and have significantly greater cytoplasm/nucleus ratio. In addition, CD56 is essential to positively identify NK cells. A previous report did not observe any CD56(+) NK cells in NLF [31], but these studies used a significantly different study population (allergic rhinitics) and were focused on IL-4 producing lymphocytes, not NK cells, within the NLF [31]. In addition, they, as well as others [5-8], perform nasal lavages by administering a single bolus dose of saline which is held in the nasal cavity and then passively expelled [31]. This is in contrast to our method, which uses repetitive spraying of smaller volumes of saline followed by forceful expulsion and collection. We speculate that compared to the bolus method of NLF collection, our method may be more mechanically disruptive to the nasal mucosa and thus produces higher numbers of NLF immune cells. However, in the NLF analyzed here, neutrophils, characterized both by cellular differential and flow cytometry analysis, were at levels similar to what has been described in bolus nasal lavages of normal human subjects at baseline by cell differential analysis [32].

Our data also show that NLF NK cells are activated during an influenza infection in healthy nonsmokers. Multiple signals contribute to NK cell activation including direct engagement of NK cell activating receptors by influenza virus [33], autocrine stimulation by activated NK cell derived chemokines [15,34], and paracrine stimulation by other immune cells like DCs [35,36]. Therefore, NK cell activation is in part dependent on communication with other cell types. We have shown here that LAIV increases cytotoxic CD16(+) NK cell percentages and granzyme B activity in the NLF of nonsmokers, but did not observe similar increases in peripheral NK cells alone. This suggests that in the setting of infection, chemokines and mediators released by other cells within the nasal mucosa assist in activating and maturing NK cells. We have previously shown that influenza-induced IP-10 levels are reduced in the nasal epithelium of smokers both *in vivo *[2] and *ex vivo *[37]. IP-10 secreted from the nasal epithelium can induce chemotaxis and enhance cytotoxic activity in resting NK cells [15], suggesting that a decrease in IP-10 levels in the context of a viral infection may lead to the suppressed NK cell cytotoxic responses demonstrated here. Tobacco smoke-induced decreases in activating cytokines such as IP-10 could create a microenvironment unfavorable for NK cell maturation resulting in suppressed CD16(+) NK cell percentages, granzyme B activity, and suppression of IFNγ in the NLF, which we have shown previously [2].

Our data suggest that peripheral NK cell responses are not affected by smoking status. CD56^bright ^CD16(-) cytokine-secreting NK cells predominate in lymph nodes and mucosal tissues, whereas CD56^dim ^CD16(+) cytotoxic NK cells are found in higher percentages in the peripheral blood [10]. Because total NK cell percentages in NLF did not change following LAIV in either nonsmokers or smokers in our study, it is possible that the cytokine milieu within the nasal mucosa induced NK cell switching from a cytokine secreting to a cytotoxic phenotype [38]. We did not observe this class switching in peripheral NK cells inoculated with LAIV, which may be due to either differences in NK cell phenotypes or the lack of maturation cytokines from exogenous immune and respiratory cells present in the nasal mucosa. In addition, while stimulation with LAIV *ex vivo *did enhance activation markers NKG2D and CXCR3 on peripheral blood NK cells in both nonsmokers and smokers, LAIV did not induce granzyme B bioactivity in peripheral NK cells *ex vivo *in either group. It is possible that peripheral and mucosal NK cell phenotypes and responses are distinct and that NK cells require a combination of signals derived from direct infection and exposure to a maturation cytokine mixture to become fully activated and secrete cytotoxic granzymes. However, differences between *in vivo *and *in vitro *LAIV inoculation procedures could account for the disparate responses between mucosal and peripheral blood NK cells. *In vivo*, LAIV infects multiple cell types including nasal epithelial cells and neutrophils in addition to NK cells. LAIV inoculation procedures *in vivo *also differ with respect to dose and timing compared to the *in vitro *inoculation. Thus although there appear to be differences between peripheral and mucosal NK cell responses, limitations in the *in vitro *study design could impact these conclusions.

Smokers are prone to respiratory microbial and viral infections, including pneumococcal pneumonia, legionellosis, meningococcal disease, rhinovirus, and influenza virus [39]. We have shown here that smokers have decreased NK cell activity in the nasal passage, and this lack of functional NK cells patrolling the upper airways may contribute to increased respiratory infections. A potential limitation of the NLF data shown here are the subtle yet statistically significant magnitude of differences in responses between nonsmokers and smokers. However, considering the interplay NK cells have with other cells of the respiratory mucosa (epithelial cells, dendritic cells), it is likely that changes in NK cell activity that appear subtle based on magnitudes of the experimental parameters could have a significant impact on overall mucosal immune responses. Interestingly, decreased NK cell cytotoxicity may also play a role in tumorigenesis in the respiratory system [40]. NK cells from smokers have decreased anti-tumor action *in vitro *[41], and decreased peripheral lymphocyte cytotoxicity *in vitro *is associated with increased cancer risk [42]. Thus, enhancement of NK cell function against infected cells or tumors could be an important therapeutic strategy for both smokers and cancer patients. For cancer patients, several NK cell therapies are already in clinical trials [43]. Adiponectin treatment of NK cells exposed to cigarette smoke *in vitro *partially restores NK cell cytotoxicity, suggesting that adiponectin may be an intriguing candidate for NK cell enhancement [44]. Thus, suppressed NK cell activity in the nasal secretions of smokers may contribute to the suppressed anti-viral and anti-tumor function seen in the respiratory tracts of smokers.

NK cells in the nasal secretions may play important roles in nasal immunity through control of respiratory viral infections both in normal individuals and those with underlying respiratory conditions. Viral infections and inflammation within the nasal passages could affect immune responses in the lower airways, especially in individuals with underlying lower airway disease such as chronic obstructive pulmonary disease (COPD) or asthma. COPD is also associated with nasal inflammation and blockage of the upper airways [45]. In asthmatic individuals, treatment of rhinitis and sinusitis improves asthma disease symptoms [46], indicating that nasal inflammation can affect asthma symptoms. Infections with viruses such as human rhinovirus are a major cause of both COPD and asthma exacerbations and the majority of these infections originate in the nose [47]. Thus, innate immune cells, particularly NK cells, could play important roles in controlling viral infections and inflammation within the nose and prevent worsening of preexisting respiratory conditions.

## Conclusions

In summary, we have demonstrated that NK cells are present in nasal secretions. NK cells could play an important role in nasal innate immunity to viruses, as well as in the suppressed immune responses to respiratory infection seen in smokers. Further study of this unique mucosal immune cell population will be beneficial in assessing the effects of both pollutants and pathogens on upper respiratory immune responses in healthy and diseased populations.

## List of Abbreviations and Symbols

α: alpha; ANOVA: analysis of variance; AUC: area under the curve; BAL: bronchoalveolar lavage; β: beta; BMI: body mass index; CCR7: c-c chemokine receptor 7; CD: cluster of differentiation; COPD: chronic obstructive pulmonary disease; CS: cigarette smoke; CSE: cigarette smoke extract; CXCL: chemokine motif ligand; CXCR3: chemokine receptor for IP-10; DC: dendritic cell; DTT: dithiothreitol; ELISA: enzyme-linked immunosorbent assay; FBS: fetal bovine serum; FITC: fluorescein isothiocyanate; FSC: forward scatter; γ: gamma; H&E: hematoxylin and eosin; HA: hemagglutinin; HAU: hemagglutinin units; HBSS: hank's buffered salt solution; HRP: horse radish peroxidase; IFN: interferons; IL: Interleukin; IP-10: interferon gamma-induced protein 10 kDa; LAIV: live attenuated influenza virus; MDCK: Madin-Darby canine kidney; NK: natural killer; NKG2D: NK cell activating receptor 2D; NLF: nasal lavage fluid; PBMC: peripheral blood mononuclear cells; PE: phycoerythrin; RANTES: regulated upon activation, normal T cell expressed and secreted; RBC: red blood cells; SEM: standard error of the mean; SSC: side scatter; TBS: tris buffered saline; α: alpha.

## Competing interests

The authors declare that they have no competing interests.

## Authors' contributions

All authors have read and approved the final manuscript. Study conception, protocol design, clinical support, and assay development: KMH, TLN, MH, and IJ; Data analysis: KMH, HZ, and HZ; Data interpretation, manuscript preparation and final approval: KMH, TLN, and IJ
